# The Role of Family Planning in Enhancing Community Resilience: Insights from Drought-Affected Youths and Women in Ethiopia

**DOI:** 10.3390/ijerph22010053

**Published:** 2025-01-01

**Authors:** Muluken Dessalegn Muluneh, Woldu Kidane, Sintayehu Abebe, Virginia Stulz, Misrak Makonnen, Makida Berhan

**Affiliations:** 1 Amref Health Africa in Ethiopia, Bole Sub City, Woreda 03, P.O. Box 20855, Addis Ababa 1000, Ethiopia; woldu.kidane@amref.org (W.K.); sintayehumoh@gmail.com (S.A.); makida.berhan@amref.org (M.B.); 2 Melbourne School of Population & Global Health, Melbourne University, Carlton, VIC 3053, Australia; 3 Faculty of Health, University of Canberra, 11 Kirinari St, Bruce, ACT 2617, Australia; virginia.stulz@canberra.edu.au

**Keywords:** family planning, resilience building, drought-affected and food-insecure regions of Ethiopia

## Abstract

This study assesses the role of family planning (FP) in resilience building among youths and women in Ethiopia’s drought-affected and food-insecure regions. A mixed-method comparative cross-sectional study design with a total of 1712 study participants with an equal 1:1 ratio of the intervention and control groups was used. Binary logistic regression analysis was carried out to identify factors associated with community resilience. More participants in the intervention districts (58.3%) than in the control districts (49%) were currently using FP services. Approximately 54.1% of the intervention group and 43.6% of the control group were able to pay for food and shelter. Two-thirds of the intervention respondents and half of the control respondents had good social cohesion. The food consumption scores for the intervention and control districts were 26.2 and 24.9, respectively. Additionally, 62.5% of the intervention and 53.5% of the control respondents were found to have a good level of community level resilience. FP use (AOR = 3.4, 95% CI: 1.78–6.49), good social cohesion (AOR = 7.9, 95% CI: 6.09–10.23) and productive assets (AOR = 1.4, 95% CI: 1.07–1.92) were significantly associated with community resilience. To enhance community resilience comprehensively, expanding FP services should empower women in decision-making processes, cultivate ties and promote collaborative efforts across different sectors.

## 1. Introduction

Globally, population pressures are massive as the world population reaches 8 billion in 2024 and is expected to reach 9.8 billion in 2050 and 11.2 billion in 2100. This growth is expected to be driven mainly by increases in less developed regions, particularly in Africa. Africa’s population is predicted to grow significantly, from approximately 1.3 billion in 2020 to 2.5 billion in 2050 [1], potentially reaching 3.9 billion by 2100 [2]. Similarly, Ethiopia’s population has grown over the last four decades from 28.3 million in 1970 to 129 million in 2024 [3]. Ethiopia, Africa’s second most populous country, needs help with rapid population growth, which places significant pressure on resources, healthcare and infrastructure [4]. This continued population growth has been identified as a global problem and is incredibly challenging in drought-prone areas, where it exacerbates food insecurity and increases economic vulnerability [5].

Resilience is crucial for maintaining livelihoods, ensuring food security and promoting the overall well-being of human beings [6,7,8,9].

Resilience can be defined as the capacity of individuals, households or communities to withstand and adapt to shocks and stressors, including environmental ones, while maintaining essential functions and expanding their adaptability [10,11].

Resilience-building efforts help to strengthen access to education, healthcare, economic opportunities, household economic stability, improve social cohesiveness, women’s decision-making capacity and food security and reduce gender violence [12,13]. For youths and women, resilience boosts their ability to handle immediate hardships and fosters long-term stability and prosperity for their families and communities [14,15].

A stable income and financial security help families better withstand and recover from natural disasters and economic downturns [16]. A 2023 study reported that the COVID-19 pandemic caused financial stress in families, leading to feelings of fear and demotivation, emphasizing the need for financial resilience to mitigate such stress [17]. Studies show that households with diversified income sources and savings recover better from shocks [18]. In Ethiopia, families involved in activities such as farming and small businesses display greater resilience during droughts [19,20]. In addition, social cohesion, or strong community relationships, improve resilience by encouraging collaborative action, mutual support, and effective crisis communication [21,22]. In conflict-affected areas, strong social networks empower communities to access resources and support systems, assisting with rehabilitation and rebuilding efforts [23]. Furthermore, different studies have shown that better food security ensures reliable access to sufficient safe, and nutritious food, helping individuals and communities maintain health and cope with crises [24,25,26,27]. Stable income, food security, proper water management, education and gender equality significantly enhance communities’ ability to adapt and build resilience [27,28]. On the other hand, family planning (FP) interventions are essential for managing population growth and improving resilience by easing demographic pressures and supporting more effective resource use [29].

FP has been recognized as a vital strategy for managing fast-growing populations and their associated challenges [30]. FP significantly contributes to community resilience by enhancing health, economic stability and environmental sustainability [31]. FP empowers individuals to plan and space pregnancies, which can lead to improved maternal and child health outcomes by reducing the risks associated with closely spaced pregnancies, high fertility rates and early childbearing [32]. This, in turn, contributes to better health, education and economic opportunities for women and their families [33,34].

Furthermore, FP plays a direct role in reducing population pressures and mitigating strains on resources, including access to clean water, food security and primary healthcare [35]. By enabling individuals to make choices that align with their desired family size and spacing, FP can assist in slowing population growth rates, providing an opportunity for sustainable socioeconomic development. Evidence shows that FP can contribute to stabilizing fast-growing populations, which in turn supports food security and nutrition-focused programs [35]. Despite massive investments by governmental and nongovernmental organizations, the utilization of modern FP, especially in developing countries, remains low.

Globally, among the 1.9 billion women of reproductive age (15–49 years) in 2019, 1.1 billion needed FP; of these, 842 million used contraceptive methods, and 270 million had an unmet need for contraception [36]. A pooled estimate of modern contraception use in sub–Saharan African countries is 18.36% [37]. In 2012 and 2017, the [38] prevalence of modern contraceptive use among married women or those in relationships in Africa was reported to be low: estimated at 23.9% and 28.5%, respectively [38,39,40,41]. A recent large population-based study to estimate the prevalence and factors associated with modern contraceptive use among women of reproductive age in 20 African countries reported a 26% prevalence of modern contraceptive use, with a country-specific variation of 6% in Guinea to 62% in Zimbabwe [42]. The 2019 Ethiopian Demographic Health Survey (EDHS) reported that the modern contraceptive prevalence rate (CPR) among currently married women aged 15–49 years was 41%, and only 1% used a traditional method. Data show that 22% of currently married women have an unmet need for FP services, 13% for spacing pregnancies and 9% for limiting the number of children [43]. Studies carried out in Ethiopian pastoralist areas revealed that 47% of women did not use FP and women who did not use FP were more likely to have shorter spacing between births, lack their partner’s support, not be involved in decisions regarding large household purchases and have low household expenditures [44]. Different studies have shown that limited access to FP leads to unwanted pregnancies, high demographic pressure, economic burdens on households and limited women’s participation in the workforce, which affect community resilience to different stressors [38,41,45,46,47].

To tackle the challenges of low FP service utilization, and poor awareness of its connection to resilience and demographic pressure, various projects have been implemented. However, evidence linking FP to resilience, particularly in drought-prone and pastoralist communities, is limited. This study aims to investigate the role of FP in enhancing community resilience and related factors among youth and women in Ethiopia’s drought-prone and chronically food-insecure regions.

## 2. Materials and Methods

### 2.1. Study Design, Setting and Population

The study used a mixed-methods comparative cross-sectional design that combined qualitative and quantitative data. Employing a comparative cross-sectional design enhances the analytical capabilities of our analysis by allowing for the exploration of differences between groups and interactions among variables [48]. Four regional states of Ethiopia, namely, Amhara (Wahigimra Zone), Oromia (Borena Zone), Afar (Zone one) and southern Ethiopia (South Omo and Wolaita Zones), were examined. The geographical areas commonly face natural and artificial disasters, and these regions are highly drought prone and have high fertility rates. Additionally, Afar and South Omo have pastoralist communities. The areas are characterized by low FP uptake and a low awareness of the nexus between FP and resilience, and demographic pressure and gender inequity are major challenges in these areas. In response to these issues, the Scaling up Family Planning for Resilience Building (RESET Plus) project was introduced, which was funded by the European Union (EU) and implemented by Amref Health Africa from December 2021 to December 2024 in five clusters/sixteen woredas [49]. The intervention areas comprised two zones in southern Ethiopia, Wolaita and South Omo, one zone in Oromia (Borena) and one zone in Amhara and the Afar region. The study districts were Duguna Fango, Boloso Bombe, Dhas, Wachile, Sekota Zuria, Abergele Hammer and Bena. Overall, the project aimed to strengthen the resilience of communities through better FP and gender equality while reducing demographic pressure. The study also included a control group composed of six districts, which were selected in the same region and zone to serve as comparators to the intervention areas. To better isolate the impact of the project, these control districts received only standard government support for FP. It was assumed that all other characteristics of the districts were similar due to random assignment, with the exception of individual FP interventions funded by the EU. The project’s FP services involve increasing the demand of the community and availability of both short- and long-acting family planning commodities at health facilities, as well as enhancing the capacity of service providers to deliver these family planning services effectively. The overall key approaches for family planning focus on enhancing community awareness and engagement by strengthening knowledge of family planning methods and promoting awareness through local leaders and organizations. They emphasize ensuring sustainable access to sexual and reproductive health (SRH) and FP services while enhancing the capacity of health facilities and providers. Additionally, these approaches aim to empower women and youths by fostering self-reliance and encouraging their participation in decision-making processes related to family planning. There are various means for education about family planning from various influences in the community, communication from printed out materials and media and education at health facilities and other community gatherings.

These areas were chosen because of their diverse challenges and traditional ways of life. The study’s source comprised youth and women living in drought-prone and chronically food-insecure regions of Ethiopia. This included both the intervention and control groups. The study unit, on the other hand, consisted of selected youth and women living in the same regions, specifically in selected districts in each zone and region.

### 2.2. Sample Size Determination and Sampling Procedure

Our study aims to compare two distinct populations, focusing on both the intervention and control groups, as our design is a comparative cross-sectional study. As a result, we used the double population proportion formula to estimate the sample size, assuming a 95% confidence level, 80% power of the study, a 5% nonresponse rate and a design effect of 2 for the multistage cluster sampling design. A 10% improvement in the utilization of FP/health and education dimensions of resilience before the project implementation (baseline evaluation) and an assumed improvement of 60% after the project implementation (end line evaluation) were assumed. It was assumed that the baseline/midterm evaluation would show a 50% improvement. Based on these assumptions and calculations, the minimum required sample size was determined to be 1712 respondents. This sample size was distributed between 856 respondents in the intervention group and 856 respondents (1:1 ratio) in the comparison group across the sampled districts. All young people and women living in drought-prone and chronically food-insecure regions who participated in FP within resilience-building programs were assigned to the intervention group. Conversely, all young people and women residing in drought-prone and chronically food-insecure areas who did not participate in FP in the intervention districts were included in the control group.

### 2.3. Sampling Procedure and Technique

Multistage sampling techniques [43] were employed to select intervention and control districts, and systematic sampling procedures were employed to reach study participants for whom actual data were collected. The women of reproductive age were identified from the sampling frame, which was generated through the assistance of health extension workers stationed at nearby health posts. A systematic approach was employed to select women from the registered sampling frame. For the qualitative part of the study, a purposive sampling procedure was used to obtain Focused Discussions (FGDs) and key informant interview (KII) study participants. For the quantitative study, the study team reached out to households and interviewed FP beneficiaries. An unstructured KII and FGD guide was used for qualitative data collection. The KIIs and FGDs targeted different stakeholders, including beneficiaries and project implementers, at different levels of project coordination.

### 2.4. Measurement

Community resilience was measured via a standard tool adapted from the American Red Cross community resilience assessment tool using the mean score [50]. Those who scored below the mean were considered to have poor community-level resilience, and those who scored above the mean were considered to have good community-level resilience.

Women’s decision-making and autonomy and gender-based violence (GBV) questions were adapted from demographic and health survey questionnaires, and the results were measured via a composite mean score [51]. Those who scored below the mean were considered to have poor decision-making skills, and those who scored the mean and above were considered to have good decision-making.

Social cohesion: This section of the tool was adapted from the well-known World Bank Social Capital and Social Cohesion tool [52]. It is measured by five items, each with a five-point Likert scale ranging from strongly disagree = one to strongly agree = five. Each item was analyzed for frequency, and a composite score was also created using the mean score. Those who scored below the mean were considered to have poor social cohesion, and those who scored the mean and above were considered to have good social cohesion.

Food consumption: This study also assessed food consumption status; the calculation followed the recommendation of the World Food Program (WFP). The food consumption score was calculated as a composite score based on dietary diversity, food frequency and the relative nutritional importance of different food groups at the household level.

Key processes within the research.

Using the research conceptual framework, the study underwent a rigorous process involving close consultation with stakeholders and beneficiaries, structured around six phases:

1. Preparation: A stakeholder meeting was held to align the program and study teams on the assessment methodologies being used. Data collection tools were developed, and data collectors were trained and piloted.
2. Desk Review: Key literature on service coverage was reviewed, focusing on resilience-friendly services and conditions.
3. Interviews: Key informant interviews were conducted with stakeholders in resilience and family planning.
4. Community Data Collection: Mixed methods were used, including interviews with women and key informants and focus group discussions.
5. Analysis and Reporting: Data cleaning and analysis were performed to synthesize our findings.
6. Stakeholder Workshop: A national workshop reviewed findings and established initial action priorities, outlining a context-specific plan to address gaps in FP.

### 2.5. Data Collection and Data Quality Management

The questionnaire was developed by reviewing the relevant literature [43,44,50,52,53]. To ensure the completeness of the data, face–to–face interviews were conducted with eligible individuals. The study participants were assigned identification numbers, which were used to distinguish between the intervention group and the control group. The identification number was used to determine whether a participant was an intervention participant or a control before data collection. The quantitative data were collected electronically via mobile devices such as KoboCollect, where a structured questionnaire with pre-coded answers was uploaded. To ensure unbiased data collection, the data collectors were intentionally blinded to the status of the respondents, preventing them from identifying the participants as intervention participants or controls.

The tool was first prepared in English and later translated into each local language in each region. To ensure consistency, the translated local version of the tool was then translated back into English. Data collectors were selected based on their experience with electronic data collection and their ability to speak the local language at the specific study sites. Before the actual data collection began, a pretest was conducted on 5% of the sample, which helped refine and contextualize the tools. Through the data collection process, the supervisor checked for the completeness and consistency of the collected data.

### 2.6. Statistical Methods

Both descriptive and inferential data analysis techniques were employed to analyze the data collected using qualitative and quantitative tools in STATA version 18 StataCorp LLC, College Station, TX, USA. Binary logistic regression analysis was carried out to identify factors associated with the dependent and independent variables. Those variables with *p* values ≤ 0.2 from the bivariable logistic regression were entered into the multivariable logistic regression to control for the possible effects of confounders, and those variables with *p* values ≤ 0.05 in the multivariable logistic regression model were considered statistically significant. Qualitative data were analyzed thematically via AtlasTi. The results were narrated and triangulated with the respective dimensions.

## 3. Results

### 3.1. Demographic Characteristics of the Respondents

In this study, there was a 98.5% response rate and data were collected from 861 (51.0%) respondents in the intervention districts and 826 (48.9%) respondents in the control district clusters. The most prominent characteristics among the respondents from both the intervention and control clusters were being Protestant, being married and having a nonformal education. In the intervention cluster, 645 (74.9%) of the mothers were currently working, whereas in the control cluster, 548 (66.3%) mothers were working. The most commonly used source of health information in both the intervention, 766 (89.0%), and control, 616 (74.6%), clusters was health extension workers, with a notable difference between the two clusters. The sociodemographic characteristics are shown in Table 1.

### 3.2. Fertility and FP Utilization

The average age of the study participants was 33 years (SD ± 7 years). The mean ages for the intervention and control clusters were 32.8 years and 32.1 years, respectively. The interventions focused on improving FP utilization to delay and/or space pregnancies and resilience building as an end goal. Consequently, the mean age at first delivery was 20 years (SD ± 3 years), with 20 years for the intervention group and 19 years for the control group. Similarly, the average family size between the intervention and control districts was five members and six members, respectively.

**Figure 1:**
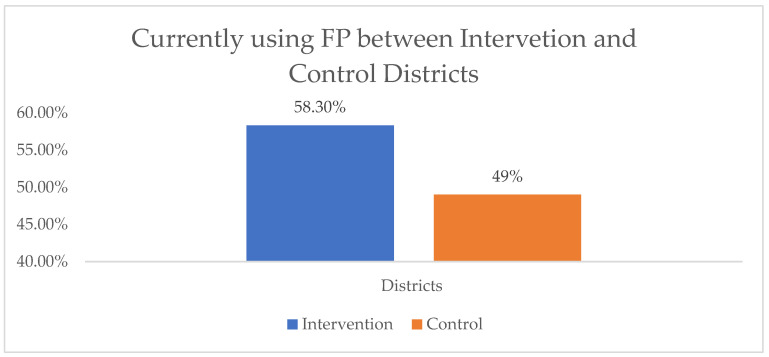
Comparison of current FP use among the intervention and control clusters, 2024.

The participants were asked about their reasons for FP use and their experience, both previously and currently. The majority of the respondents, 1527 (90.5%), believed that pregnancy planning was important for the family. Among these, 796 (92.5%) were from the intervention group, and 731 (88.5%) were from the control group (*p* = 0.006). Most of the study participants from the intervention (79.4%) and control (70.4%) clusters reported that they had used FP in their lifetime. Additionally, more participants in the intervention cluster (58.3%) than in the control group (49%) were currently using FP. A comparison of the current use of FP between the intervention and control clusters is shown in Figure 1.

Outreach services and mobile health teams were crucial in overcoming distance and accessibility challenges, especially for communities affected by drought and displacement. A quote from the MCH director of the Zonal Health Department in the Borena Zone highlighted the importance of these services: “The Borena Zone is sometimes affected by drought, and during such times, our routine services may not be available. The community may be displaced from their homes and lose their property, settling in different IDP centers. In these circumstances, it is crucial to establish mobile teams to provide services in the areas where the community is located. The support provided by the project was very beneficial in this regard”.

A successful approach to improving awareness and service utilization among youths includes offering youth-friendly services and recreational centers. These approaches have effectively promoted youth health service utilization by ensuring confidentiality. Another nongovernmental representative from the South Omo field office said that:

“It is crucial for FP services to be confidential and private, especially for youths. This requires trained health workers, setting up a separate room to access the service for the youth, counseling, and discussion. Amref Health Africa has significant experience in this regard by building and supporting youth medical and recreation centers. These centres have made young people use the services happily and without embarrassment”.

Women’s decision-making autonomy and gender-based violence (GBVs)

**Figure 2:**
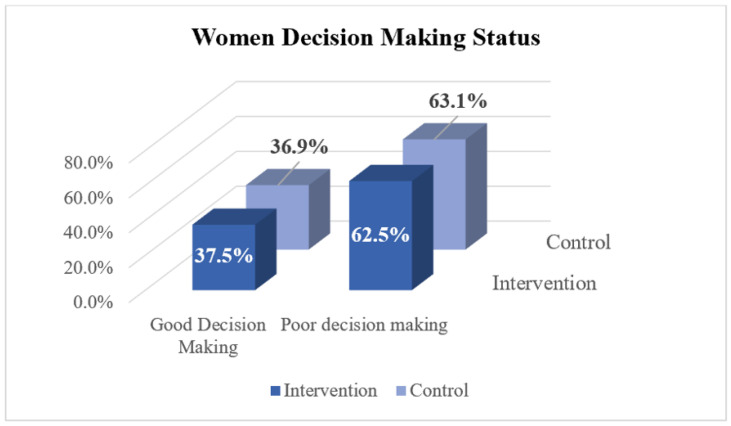
Decision-making autonomy of women in the intervention and control clusters, 2024 (*N* = 1687).

The culture in Ethiopia has historically given women at a lower status in the home, especially in regard to making decisions about healthcare, which is crucial for women’s empowerment. This study assessed women’s decision-making autonomy at the household level. The study revealed that for all five variables assessed, decisions regarding access to healthcare and household purchases were made mainly jointly by the husband and wife, with a lower proportion of decisions made solely by women. For example, in regard to decisions about major household purchases, husbands made 277 (16.4%) decisions, whereas wives made 147 (8.7%) decisions. Additionally, the study compared women’s decision-making autonomy between the intervention and control clusters but found no significant difference (*p* = 0.82), as shown in Figure 2.

Some study participants mentioned that women’s decision-making ability is closely linked to economic empowerment. They emphasized that empowering women economically not only improves their decision-making ability but also enhances household resilience. One nongovernmental representative from the Waghimra Field Office stated:

“Most of the time, women are economically dependent on their husbands, which hinders their access to reproductive health services. However, when women generate income, they are economically empowered, which benefits their ability to access health services”.

In this study, various forms of GBV were assessed. The results revealed that a significant number of participants reported experiences of controlling behavior, jealousy, physical violence and sexual violence by their husbands or partners. Specifically, 72.1% of the participants reported that their husband or partner insisted on always knowing their whereabouts. Additionally, 30.5% reported that their husband or partner became jealous or angry if they talked to other men. Furthermore, 12.3% experienced physical violence, such as being kicked, dragged or beaten, whereas 19.6% reported experiencing sexual violence. Among those who experienced sexual violence, 13.9% were from intervention clusters, and 14.1% were from control clusters. It was reported that their husband or partner physically forced them to have sexual intercourse with them. GBV among the study participants is shown in Table 2.

In this study, the researchers investigated whether the participants justified beating or not. It was found that 29.5% of the participants seemed to justify beating. The study also revealed that justification decreased as participants moved from rural areas to urban areas. Furthermore, the participants were asked about their reasons for justifying beating, with the following responses: burning food (21.3%), going out without informing their husbands (11.9%), arguing with their husbands and refusing to have sexual intercourse with their husbands (4.6%).

### 3.3. Household Economic Stability and Livelihoods

The study evaluated the economic stability of the participants at the household level. Approximately 54.1% of the respondents from the intervention group and 43.6% from the control group mentioned that they struggled to make lump-sum payments for health and education expenses, despite being able to pay for food and shelter. Additionally, a significant number of participants (23.5% from the intervention group and 29.1% from the control group) stated that they would rely on other income-generating activities or family support if they were to lose their primary source of income.

The study also examined whether households owned liquid assets that could be quickly converted into cash, such as livestock, food stores or personal belongings. A considerable percentage of participants (24.0% from the intervention group and 27.4% from the control group) reported that they did not possess many liquid assets. Furthermore, the availability of productive assets was assessed, with over half of the respondents (53.1% from the intervention group and 52.1% from the control group) indicating that they had few productive assets. Household economic stability and livelihood are shown in Table 3.

The qualitative study supports the finding that economic empowerment was a successful strategy within the project. Notably, involving women in income-generating activities improves their economic status and independence, which in turn enhances their access to health services, including reproductive health, and reduces marital dominance. The Maternal, Child Health and Nutrition Coordinator from Wolaita Zone said:

“For young people in Areka, a Digital Satellite Television (DSTV) entertainment house was opened to prevent them from spending time in bad places. This allowed them to watch football matches, increase their income, and build resilience”.

### 3.4. Social Cohesion

This research examined the level of connection and unity within a community, as well as the shared resources available to its members [54]. Among the 180 (20.9%) participants from the intervention group and 165 (19.9%) from the control group, a significant number reported feeling very close to their social network. When asked about their involvement in community meetings to address local issues, a significant proportion of participants (41.9%, *p* < 0.0001) stated that they participated regularly. Additionally, when questioned about how their community tackles problems, 35.6% said that the entire community works together to solve issues, whereas 33% stated that individuals within each social group come together to address problems.

**Figure 3:**
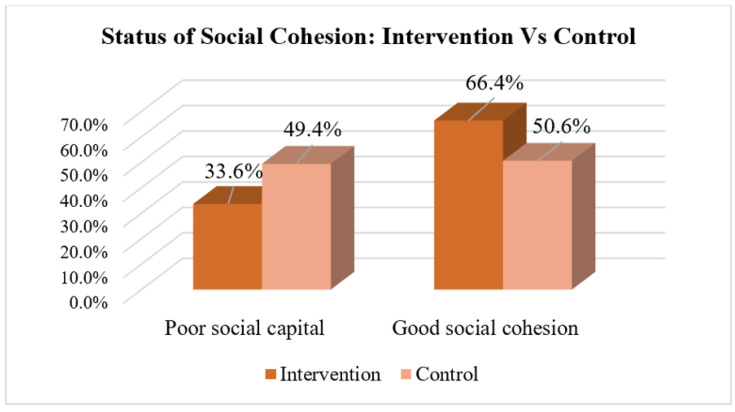
Comparison of social cohesion among the intervention and control clusters, 2024, *N* = 1687.

The findings of the World Bank Social Capital and Social Cohesion Tool revealed that, compared with 418 (50.6%) control districts, 572 (66.4%) intervention districts demonstrated a relatively good rate of social cohesion among their communities (*p* < 0.0001). A comparison of social cohesion between the intervention and control clusters is shown in Figure 3.

### 3.5. Food Security

This study evaluated food security via the household-level food insecurity scale (HFIAS) from USAID and the food consumption scale from the World Bank. Overall, there was a slight improvement in the intervention clusters for most of the variables assessed in comparison with those in the control group. The findings show the inability to eat preferred food types due to a lack of resources (72.2% vs. 74.2%), eating fewer meals in a day due to a lack of food (51.6% vs. 55.1%), going to sleep hungry at night (24.9% vs. 28.3%) and going a whole day and night without eating (18.0% vs. 25.4%) in the intervention and control clusters, respectively. Table 4 shows food security at the household level between the intervention and control clusters.

### 3.6. Community Resilience/Capacity to Manage Shock

This study compared the level of community resilience between the intervention and control clusters. In total, 538 (62.5%) participants from the intervention cluster and 442 (53.5%) from the control cluster had a good level of community resilience (*p* value < 0.0001). Table 5 shows the community resilience level.

### 3.7. Association of Variables with Community-Level Resilience

Binary logistic regression was used to identify the variables associated with community-level resilience, which was measured via a standard validated tool. In the bivariable logistic regression analysis, variables with a *p* value less than 0.2 were considered. In the multivariable logistic regression analysis, the following variables remained statistically significant with respect to community-level resilience: FP use, good decision-making power, good social cohesion and the availability of productive assets.

Accordingly, a community that uses FP was 3.4 times (AOR = 3.4, 95% CI: 1.78–6.49) more likely to be resilient than those who did not use FP. Compared with the control group, the intervention group was highly likely (AOR = 1.3, 95% CI: 1.01–1.66) to be resilient. Similarly, a community that possesses good decision-making abilities (AOR = 1.9, 95% CI: 1.23–2.65) was more likely to be resilient than those that did not have good decision-making abilities (Table 6).

Social cohesion was assessed, and communities that possessed good social cohesion were more likely to be resilient than their counterparts were 7.9 (AOR = 7.9, 95% CI: 6.09–10.23). Those who have productive assets (AOR = 1.4, 95% CI: 1.07–1.92) were also 1.4 times more resilient compared with those who did not have productive assets at all.

## 4. Discussion

This study provides valuable insights into the resilience of youths and women in drought-prone and chronically food-insecure regions of Ethiopia. By comparing a post-intervention group with a control group, we found significant differences in community-level resilience, with 62.5% of the intervention group demonstrating a good level of resilience compared with 53.5% of the control group. Notably, the intervention group was 1.3 times more likely to exhibit resilience, highlighting the effectiveness of the multifaceted approach implemented in this study. This can be explained by the project’s multifaceted intervention, which included FP awareness creation for the community, improved-quality SRH services, economic empowerment initiatives and multisectoral engagement. These components collectively enhanced the community’s ability to effectively manage resources, access quality healthcare and participate in economic activities, ultimately contributing to greater resilience.

The results of our study revealed a statistically significant connection between the use of FP, strong decision-making authority, robust social cohesion and the availability of productive assets with community-level resilience. A community that uses FP is three times more likely to be resilient than a community that does not use FP. This finding is supported by a study conducted in Tanzania [55]. A 2023 brief discussed an intervention in Niger, showing that family planning utilization and community resilience are statically significant [56]. Like our study, this study reflects the fact that the long-term benefits of FP on health and economic stability can significantly contribute to building resilient communities capable of adapting to environmental and socio-economic changes. This can be explained by the fact that FP enables families to space and limit births. FP improves maternal and child health by allowing for better birth spacing and timing, which directly contribute to healthier families and communities. This health improvement is essential for resilience, especially in the face of environmental and socio-economic challenges. This helps them manage resources more effectively, invest in education and health and reduce financial strain, all of which contribute to greater resilience [40,57].

The study findings show that women with significant decision-making autonomy are twice as likely to exhibit resilience than those without such autonomy [58]. Particularly in areas such as accessing healthcare and purchasing household goods, women’s decision-making autonomy at the household level has a noteworthy impact on resilience. However, our findings indicate that decisions made solely by women are less common than joint decisions made by both spouses. This is likely due to traditional gender roles and cultural norms that prioritize joint or male-dominated decision-making within households. These norms can restrict women’s ability to independently make important decisions that affect their well-being and the resilience of their families. Empowering women to make autonomous decisions can result in improved health outcomes, better resource management and increased economic stability, ultimately contributing to greater overall resilience [53,58,59,60].

Social cohesion was assessed, and communities that possess good social cohesion were eight times more likely to be resilient than their counterparts. This finding can be explained by the fact that strong social cohesion creates a supportive environment where community members actively participate in collective problem solving and resource sharing. When individuals feel connected and supported by their social network, they are more likely to collaborate, share resources and help one another during times of need. This collective approach enhances the community’s overall ability to adapt to and recover from adverse conditions, thereby significantly increasing resilience [61].

Moreover, women who have productive assets are 1.4 times more likely to be resilient than those who do not have productive assets at all. This finding is supported by various studies and reports that highlight the positive correlation between women’s ownership of productive assets and their resilience [62,63,64,65]. This can be explained by the fact that productive assets, such as land, livestock, equipment or small businesses, provide a stable source of income and resources. These assets can be used to generate wealth, improve living conditions and act as a buffer against economic shocks. Having productive assets enables individuals and families to invest in health, education and other critical areas, enhancing their overall ability to cope with and recover from adverse conditions. Consequently, the ownership of productive assets plays a significant role in building and sustaining resilience.

## 5. Practical Significance and Future Insights of the Research

This research on the role of FP in enhancing community resilience in drought-affected regions of Ethiopia holds significant practical implications. By demonstrating that increased FP service utilization correlates with improved economic stability, social cohesion, and food security, the study provides a compelling case for integrating FP services into resilience-building initiatives. This is particularly crucial in resource-limited settings, where the pressures of rapid population growth exacerbate existing vulnerabilities. The findings suggest that empowering women through FP not only enhances their health outcomes but also enables them to make informed decisions that contribute to the well-being of their families and communities. Such insights can guide policymakers and practitioners in designing interventions that address both demographic pressures and community resilience effectively. Moreover, the study’s emphasis on the interconnectedness of FP, economic stability and social cohesion highlights the need for a multidisciplinary approach to addressing the complex challenges faced by communities in drought-prone areas. By fostering collaboration among health, agricultural and social sectors, stakeholders can create comprehensive strategies that promote sustainable development. The evidence presented in this research can be instrumental in advocating for increased investment in FP services as a critical component of resilience-building programs, ultimately leading to improved livelihoods and enhanced community well-being.

Additionally, from a scientific perspective, this research contributes to the growing body of literature linking family planning to broader socioeconomic outcomes, particularly in vulnerable populations. Furthermore, this research underscores the importance of considering cultural and environmental factors when evaluating the impacts of FP, thereby encouraging a more nuanced understanding of reproductive health in relation to community resilience. As a result, this study not only advances the field of public health but also opens avenues for interdisciplinary collaboration among social scientists, health researchers, and policymakers. Future research can build on these findings by exploring the specific mechanisms through which FP impacts resilience and by examining the long-term effects of FP initiatives on community dynamics. Additionally, longitudinal studies could provide deeper insights into how changes in FP utilization over time affect overall community resilience, thereby enhancing the scientific understanding of this critical issue.

### Limitations of the Research

This study has some limitations, including its cross-sectional design, which may not fully capture the causal relationships involved. Furthermore, the study areas were highly susceptible to emergencies such as COVID-19, drought and conflict, which significantly influenced the relationship between resilience and the effects of family planning. Additionally, difficulties in data collection at the beginning of the study impacted its potential generalizability for comparing the regions at the end of the project. Despite these challenges, the integration of quantitative and qualitative methods offers a strong foundation for understanding Ethiopia’s primary healthcare system.

## 6. Conclusions and Recommendations

Our study demonstrates that access to family planning (FP) services is linked to enhanced economic stability, improved food security, and increased social cohesion. Specifically, the intervention group—which focused on boosting community awareness and engagement by strengthening knowledge of family planning methods and promoting awareness through local leaders and organizations—saw a more than 10% increase in FP users compared to the control group, despite no additional investment in FP services. This increase was associated with significant resilience outcomes at the community level, including greater decision-making autonomy for women and improved food consumption scores. From this evidence, we can conclude that family planning is not merely a health service; it serves as a critical foundation for resilience against environmental and economic shocks. FP significantly enhances the lives of youth and women in drought-prone areas of Ethiopia. By providing access to reproductive health services and education, FP empowers individuals to make informed choices about their futures, thereby improving overall well-being. Moreover, the evidence shows that achieving abstract goals like resilience can be facilitated through effective family planning interventions. These programs not only help in managing population growth but also strengthen community stability, allowing families to better cope with the challenges posed by repeated droughts. By fostering economic opportunities and promoting health, FP contributes to building a more resilient society capable of withstanding environmental stresses.

The general state of resilience that can be seen from our evidence reveals the linking role played by FP in multiple dimensions of resilience, including economic and social cohesion. In addition, our analysis has important policy implications: it shows that FP interventions have a doubly beneficial role in crisis settings by helping to reduce population growth but also empowering women with agency over their reproductive lives and enabling families to better manage resources effectively, increasing household resilience. This reciprocal relationship highlights the importance of integrated health services and economic empowerment strategies to strengthen community resilience holistically.

To increase the positive impact of FP on resilience, key priorities for action exist across levels. One needs to scale up investments in FP services to address such the demand of the community for contraception and inequities in access in Ethiopia specially in the vulnerable population such as drought and pastoralist communities. Hence, decision-makers and program implementors at all levels, including government agencies, NGOs and international organizations, should prioritize increased funding and support for FP programs. This includes expanding access to a wide range of contraceptive methods, ensuring the availability of youth-friendly services, and addressing cultural and societal barriers to FP uptake. It is critical to prioritize the promotion of women’s decision-making power through programs that educate and engage the community while putting women first as leaders at home and in their communities. Activities that improve social cohesion by building a network of mutual support are crucial for resilience. An integrated approach to FP and economic empowerment will help families better manage their resources and support informed decision-making. Finally, the promotion of multisectoral collaboration between governmental and nongovernmental groups will enable holistic support systems to confront the complex burdens these communities face. In this way, decision-makers can go a long way to substantially strengthening the resilience of vulnerable people, whether in Ethiopia or elsewhere.

## Figures and Tables

**Table 1:** Characteristics of respondents disaggregated by intervention and control clusters, 2024.

Variables	Study Participants (*N* = 1687)	Intervention (*N* = 861)	Control (*N* = 826)
Age	*n* (%)	*n* (%)	*n* (%)
15–19 years	53 (3.1)	25 (2.9%)	28 (3.4%)
20–29 years	563 (33.4)	272 (31.6%)	291 (35.2%)
30–39 years	763 (45.2)	400 (46.5%)	363 (43.9%)
40–49 years	308 (18.3)	164 (19.0%)	144 (17.4%)
Zones/Cluster			
Wolaita	745 (44.2)	347 (20.6%)	398 (23.6%)
Waghimra	354 (20.9)	247 (14.6%)	107 (6.3%)
Borana	272 (16.5)	70 (4.2%)	202 (11.9%)
South-Omo	316 (18.7)	197 (11.7%)	119 (7.1%)
Place of residence			
Rural	1058 (62.7)	467 (54.3%)	591 (71.5%)
Urban	629 (37.3)	394 (45.7%)	235 (28.4%)
Religion			
Protestant	659 (39.1)	330 (38.3%)	329 (39.8%)
Orthodox Christian	539 (31.9)	293 (34.0%)	246 (29.8%)
Traditional	404 (23.9)	220 (25.6%)	184 (22.3%)
Muslim	70 (4.2)	11 (0.7%)	59 (7.1%)
Catholic	15 (0.9)	7 (0.8%)	8 (1.0%)
Marital status			
Married	1474 (87.4)	739 (85.8%)	735 (89.0%)
Widowed	113 (6.7)	62 (7.2%)	51 (6.2%)
Divorced	61 (3.6)	37 (4.3%)	24 (2.9%)
Single	39 (2.3)	23 (2.7%)	16 (2.9%)
Current working status			
Working	1193 (70.7)	645 (74.9%)	548 (66.3%)
Not working	494 (29.2)	216 (25.1%)	278 (33.7%)
Woman’s educational status			
No formal education	937 (55.5)	488 (56.7%)	449 (54.4%)
Primary (1–8 grade)	525 (31.1)	258 (30.0%)	258 (21.2%)
Lower secondary (9–10 grade)	105 (6.2)	57 (6.6%)	48 (5.8%)
Preparatory (11–12 grade)	55 (3.3)	14 (1.6%)	41 (5.0%)
Technical Education and Vocational Training (TEVT)	17 (1.0)	8 (0.9%)	9 (1.1%)
Higher Education	48 (2.9)	36 (4.2%)	12 (1.5%)
Partner’s educational status (*n* = 838)	(*n* = 1648)	(*n* = 838)	(*n* = 810)
No formal education	927 (56.3)	497 (59.3%)	430 (53.1%)
Primary (1–8 grade)	394 (23.9)	194 (23.2%)	200 (24.7%)
Lower secondary (9–10 grade)	132 (8.0)	61 (7.3%)	71 (8.8%)
Preparatory (11–12 grade)	51 (3.1)	18 (2.1%)	33 (4.1%)
Technical Education and Vocational Training (TEVT)	29 (1.8)	13 (1.6%)	16 (2.0%)
Higher Education	115 (6.9)	55 (6.6%)	60 (7.4%)
Common source of health information			
Health extension workers	1382 (81.8)	766 (89.0%)	616 (74.6%)
Healthcare providers	190 (11.3)	41 (4.8%)	149 (18.0%)
Radio	17 (1.0)	8 (0.9%)	5 (0.6%)
Television	13 (0.8)	3 (0.3%)	14 (1.7%)
None	85 (5.1)	43 (5.0%)	42 (5.1%)

**Table 2:** Comparison of gender-based violence between the intervention and control clusters, 2024 (*N =* 1687).

Variables (Past 12 Months)		Freq (% of Responses)	Freq (% of Cases)	Intervention (%)	Control (%)	*p* Value
Which of the following happened to you in your relationship (*n* = 2414)	Jealousy	515 (21.3)	515 (30.5)	235 (18.7)	280 (24.2)	<0.0001
	He accuses me	98 (4.1)	98 (5.8)	52 (4.1)	46 (3.9)	
	He did not want me to meet friends	217 (8.9)	217 (12.9)	114 (9.1)	103 (8.9)	
	Limit my contact with family	368 (15.2)	368 (21.8)	227 (18.1)	141 (12.2)	
	Insisted on knowing where I am	1216 (50.4)	1216 (72.1)	627 (49.9)	589 (50.8)	
My partner did the following (*n* = 1993)	Push you, shake you, or throw something at you	107 (5.4)	107 (6.3)	56 (5.6)	51 (5.2)	0.012
	Slap you	203 (10.2)	203 (12.0)	98 (9.7)	105 (10.7)	
	Twist your arm or pull your hair	48 (2.4)	48 (2.9)	17 (1.7)	31 (3.1)	
	Punch you	27 (1.3)	27 (1.6)	13 (1.3)	14 (1.4)	
	Kick you, drag you, or beat you up	208 (10.4)	208 (12.3)	128 (12.7)	80 (8.1)	
	Try to choke you or burn you on purpose	85 (4.3)	85 (5.0)	34 (3.4)	51 (5.2)	
	Threaten or attack you with a knife, gun	17 (0.9)	17 (1.0)	9 (0.9)	8 (0.8)	
	None	1298 (65.9)	1298 (76.9)	653 (62.8)	645 (65.5)	
Sexual violence experience (*n* = 1755)	Physically force you to have sexual intercourse with him	245 (13.9)	245 (14.5)	124 (13.9)	121 (14.1)	0.09
	Physically force you to perform any other sexual acts	44 (2.5)	44 (2.6)	27 (3.0)	17 (1.9)	
	Force you with threats or in any other way to perform sexual acts	42 (2.4)	42 (2.5)	21 (2.3)	21 (2.4)	
	None	1424 (81.1)	1424 (81.4)	722 (80.8)	702 (81.5)	
Emotional Violence (*n* = (1784)	Say or do something to humiliate you in front of others	106 (5.9)	106 (6.3)	63 (6.9)	43 (4.9)	0.54
	Threaten to hurt or harm you or someone close to you	46 (2.6)	46 (2.7)	23 (2.5)	23 (2.6)	
	Insult you or make you feel bad about yourself	206 (11.5)	206 (12.2)	111 (12.1)	94 (10.8)	
	None	1427 (79.9)	1427 (84.6)	719 (78.5)	708 (81.6)	

**Table 3:** Household economic stability measures, 2024 (*N* = 1687).

Variable		Freq (%)	Intervention (%)	Control (%)	*p* Value
A household’s ability to meet its basic needs, including food, shelter, education, and healthcare	Struggle to pay for food and shelter	313 (18.6)	130 (15.1)	183 (22.2)	<0.0001
	Usually, pay for food and shelter, but we struggle to make lump-sum payments for health and education expenses	826(48.9)	466 (54.1)	360 (43.6)	
	Usually, pay for food, shelter, education, and health care expenses. Sometimes we struggle, but we usually make lump-sum payments	496 (29.3)	238 (27.6)	257 (31.1)	
	Always able to pay for food, shelter, education, and health care without struggle	41 (2.4)	24 (2.8)	17 (2.1)	
	Refused to answer	12 (0.7)	3 (0.3)	9 (1.1)	
Household assets that can be turned into cash quickly, such as livestock, food stores, or personal belongings. These are called liquid assets. What describes your household best?	Never have many liquid assets	433 (25.7)	207 (24.0)	226 (27.4)	0.173
	Some liquid assets, but the amount changes a lot during the year	953 (56.5)	506 (58.8)	447 (54.1)	
	Some liquid assets and the amount change a little during the year	250 (14.8)	128 (14.9)	122 (14.8)	
	Always have many liquid assets	45 (2.7)	18 (2.1)	27 (3.3)	
	Refused to answer	6 (0.4)	2 (0.2)	4 (0.5)	
Household’s economic status	Barely surviving	393 (23.3)	170 (19.7)	223 (27.0)	0.002
	Struggling to make ends meet we are surviving, but our economic status is not stable	971 (57.6)	525 (61.0)	446 (54.0)	
	Mostly stable and we are investing in new opportunities, though we sometimes struggle	296 (17.6)	156 (18.1)	140 (16.9)	
	Not vulnerable: we are stable and secure	21 (1.2)	9 (1.0)	12 (1.5)	
	Refused to answer	6 (0.4)	1 (0.1)	5 (0.6)	
Productive assets are the resources used to generate income, like livestock, land for agriculture, tools, or equipment for a business. How would you describe your household’s productive assets?	No productive assets	424 (25.1)	194 (22.5)	230 (27.8)	0.037
	Few productive assets	887 (52.3)	457 (53.1)	430 (52.1)	
	Some productive assets	357 (21.2)	198 (23.0)	159 (19.2)	
	A lot of productive assets	14 (0.8)	10 (1.2)	4 (0.5)	
	Refused to answer	5 (0.3)	2 (0.2)	3 (0.4)	
When was the last time the household experienced a shock or emergency that had a major effect on your household finances, such as taking in new dependents, losing a wage earner, natural disaster, or losing a business?	No shocks have ever occurred	529 (31.4)	262 (30.4)	267 (32.3)	0.026
	More than 10 years ago	82 (4.9)	41 (4.8)	41 (5.0)	
	5–10 years	89 (5.3)	60 (7.0)	29 (3.5)	
	1–5 years	338 (20.0)	181 (21.0)	157 (19.0)	
	In the last year	643 (38.1)	315 (36.6)	328 (39.7)	
	Refused to answer	6 (0.4)	2 (0.2)	4 (0.5)	
What would happen if the same shock happened today?	Household would never recover	446 (26.4)	216 (25.1)	230 (27.8)	0.012
	Household would slowly recover	883 (52.3)	486 (56.4)	397 (48.1)	
	Household would quickly recover	34 (2.0)	18 (2.1)	16 (1.9)	
	Household would not be affected/would recover immediately	20 (1.2)	9 (1.0)	11 (1.3)	
	No answer	290 (17.2)	127 (14.8)	163 (19.7)	
	Refused to answer	14 (0.8)	5 (0.6)	9 (1.1)	

**Table 4:** Household security status among intervention clusters, 2024 (*N* = 1687).

Variables (Past Four Weeks)	Yes	Intervention		How Often the Access Problem Happens?			*p* Value
				Rarely	Sometimes	Often	
		*n*	%	*n* (%)	*n* (%)	*n* (%)	
Did you worry that your household would not have enough food?	Yes	571	66.3	310 (54.3)	216 (37.8)	45 (7.9)	<0.0001
Were you or any household member not able to eat the kinds of foods you preferred because of a lack of resources?	Yes	622	72.2	284 (45.7)	256 (41.2)	82 (13.2)	<0.0001
Did you or any household member have to eat a limited variety of foods due to a lack of resources?	Yes	679	78.9	302 (44.5)	266 (39.2)	111 (16.3)	<0.0001
Did you or any household member have to eat some foods that you truly did not want to eat because of a lack of resources to obtain other types of food?	Yes	534	62.0	257 (48.1)	210 (39.3)	67 (12.5)	<0.0001
Did you or any household member have to eat a smaller meal than you felt you needed because there was not enough food?	Yes	495	57.5	242 (48.9)	189 (38.2)	64 (12.9)	<0.0001
Did you or any household member have to eat fewer meals in a day because there was not enough food?	Yes	444	51.6	231 (52.0)	158 (35.6)	55 (12.4)	<0.0001
Was there ever no food to eat of any kind in your household because of lack of resources to get food?	Yes	216	25.1	133 (61.6)	61 (28.2)	22 (10.2)	<0.0001
Did you or any household member go to sleep at night hungry because there was not enough food?	Yes	214	24.9	183 (85.5)	29 (13.6)	2 (0.9)	<0.0001
Did you or any household member go a whole day and night without eating anything because there was not enough food?	Yes	155	18.0	136 (87.7)	18 (11.6)	1 (0.6)	<0.0001
Key							
	Food Secure		Mildly Insecure		Moderately Insecure		Severely Insecure

**Table 5:** Comparison of community resilience between the intervention and control clusters, 2024 (*N* = 1687).

Community Resilience Dimensions	Strongly Agree		Agree		Neutral		Disagree		Strongly Disagree		*p* Value
	Intervention *n (%)*	Control *n (%)*	Intervention *n* (%)	Control *n* (%)	Intervention *n* (%)	Control *n* (%)	Intervention *n* (%)	Control *n* (%)	Intervention *n* (%)	Control *n* (%)	
Hope about the future	21 (2.4)	76 (9.2)	128 (14.9)	139 (16.8)	68 (7.9)	131 (15.9)	509 (59.1)	386 (46.7)	135 (15.7)	94 (11.4)	<0.0001
Help each other	10 (1.2)	38 (4.6)	127(14.0)	215 (26.0)	42 (4.9)	52 (6.2)	575 (66.8)	430 (52.1)	107 (12.4)	91 (11.0)	<0.0001
Has resources needed to respond to problems	64 (7.4)	101 (12.2)	241 (27.9)	233 (28.2)	192 (22.3)	148 (17.9)	334 (38.8)	284 (34.4)	30 (3.5)	60 (7.3)	<0.0001
Able to receive the services they need	33 (3.8)	64 (7.7)	187 (21.7)	240 (29.1)	147 (17.1)	124 (15.0)	436 (50.6)	365 (44.2)	58 (6.7)	33 (3.9)	<0.0001
Know where to go to get things done	12 (1.4)	35 (4.2)	176 (20.4)	200 (24.2)	131 (15.2)	162 (19.6)	488 (56.7)	385 (46.6)	54 (6.3)	44 (5.3)	<0.0001
Works with organizations and agencies	8 (0.9)	73 (8.8)	221 (25.6)	235 (28.5)	99 (11.5)	150 (18.1)	489 (56.7)	331 (40.1)	44 (5.1)	37 (4.5)	<0.0001
Work together to improve the community	12 (1.4)	40 48.8)	197 (22.9)	208 (25.2)	85 (9.8)	125 (15.1)	503 (58.4)	404 (48.9)	64 (7.4)	49 (5.9)	<0.0001
Looks at successes and failures and learns from them	14 (1.6)	43 (5.2)	228 (26.5)	216 (26.2)	142 (16.5)	191 (23.1)	444 (51.6)	191 (23.1)	33 (3.8)	32 (3.9)	<0.0001
Develops skills, finds resources and solves problems	11 (1.3)	45 (5.4)	207 (24.0)	188 (22.8)	130 (15.1)	195 (23.6)	453 (52.6)	371 (44.9)	60 (6.9)	27 (3.3)	<0.0001
Makes priorities and sets goals for the future	18 (2.1)	42 (5.1)	225 (26.1)	184 (22.3)	145 (16.8)	210 (25.4)	443 (51.5)	368 (44.6)	30 3.5)	22 (2.7)	<0.0001
Tries to prevent disasters	14 (1.6)	38 (4.6)	188 (21.8)	150 (18.2)	111 (12.9)	178 (21.6)	516 (59.9)	432 (52.3)	32 3.7)	28 (3.4)	<0.0001
Actively prepares for future disasters	13 (1.5)	31 (3.8)	225 (26.1)	195 (23.6)	122 (14.2	223 (26.9)	470 (54.6)	358 (43.3)	31 3.6)	19 (2.3)	<0.0001
Provide emergency services during disaster	30 (3.5)	68 (8.2)	198 (22.9)	172 (20.8)	91 (10.6)	166 (20.2)	504 (58.5)	395 (47.8)	38 4.4)	25 (3.0)	<0.0001
If a disaster occurs, the community provides info.	9 (1.0)	36 (4.4)	208 (24.1)	198 (23.9)	85 (9.9)	145 (17.6)	505 (58.7)	413 (50.0)	54 (6.3)	34 (4.1)	<0.0001
I obtain information to help with my home and work life	7 (0.8)	39 (47)	209 (24.3)	197 (23.9)	72 (8.4)	135 (16.3)	497 (57.8)	413 (50.0)	76 (8.8)	42 (5.1)	<0.0001

**Table 6:** Multivariable logistic regression of community-level resilience among respondents, 2024.

Variables	Options	Frequency	AOR	95% CI	*p* Value
Ever use FP (No = 1)	Yes	1615	3.4	(1.78–6.49)	<0.0001 ***
Study area (control = 1)	Intervention	861	1.3	(1.01–1.66)	0.043 *
Marital status (widowed = 1)	Single	39	1.1	(0.42–2.93)	0.84
	Married	1474	0.9	(0.52–1.59)	0.75
	Divorced	61	0.7	(0.31–1.45)	0.32
Decision-making (poor = 1)	Good	628	1.9	(1.23–2.65)	0.001 **
Residence (Urban = 1)	Rural	1058	0.9	(0.64–1.19)	0.39
Social Cohesion (poor = 1)	Good	990	7.9	(6.09–10.23)	<0.0001 ***
Zone or cluster (Wolayta = 1)	Borena	272	1.04	(0.67–1.62)	0.84
	South Omo	316	0.4	(0.22–0.56)	<0.0001 ***
	Waghimra	354	0.6	(0.43–0.89)	0.01 **
Participating in community-level meetings (Never = 1)	Always	65	1.6	(0.72–3.64)	0.23
	Regularly	708	1.2	(0.72–3.64)	0.55
	Sometimes	396	2.4	(1.25–4.61)	0.009 **
	Very rarely	383	2.3	(1.67–4.74)	0.01 **
Closeness to the community (Very close = 1)	Do not close	11	2.8	(0.66–12.65)	0.16
	Not at all close	52	0.8	(0.37–1.65)	0.53
	Not Close	245	1.5	(0.98–2.29)	0.06
	Close	1034	0.8	(0.54–1.05)	0.09
Satisfaction at health facility (No = 1)	Yes	1205	0.81	(0.59–1.11)	0.19
Decision to go to health facility (Other person = 1)	Husband alone	102	0.49	(0.15–1.57)	0.23
	Respondent alone	223	0.66	(0.26–1.69)	0.39
	Respondent and Husband	1342	0.52	(0.20–1.32)	0.17
Decision to go to health facility for SRH care (Other person = 1)	Husband alone	93	4	(0.88–18.1)	0.07
	Respondent alone	326	2.6	(0.67–10.61)	0.17
	Respondent and Husband	1225	2.6	(0.68–10.38)	0.16
Availability of productive assets (No = 1)	Yes	1687	1.4	(1.07–1.92)	0.02 *

* *p* < 0.05 is significant, ** *p* < 0.01 highly significant and *** *p* < 0.001 very highly significant.

## Data Availability

The data presented in this study are available upon request to the corresponding author. The data are not publicly available due to privacy reasons.
